# Postoperative Chemotherapy Bladder Instillation After Radical Nephroureterectomy: Results of a European Survey from the Young Academic Urologist Urothelial Cancer Group

**DOI:** 10.1016/j.euros.2020.10.003

**Published:** 2020-11-06

**Authors:** Tom-Régis Dobé, Gianluigi Califano, Friedrich-Carl von Rundstedt, Idir Ouzaid, Simone Albisinni, Atiqullah Aziz, Ettore Di Trapani, Kees Hendricksen, Wojciech Krajewski, Andrea Mari, Marco Moschini, Andrea Necchi, Aidan P. Noon, Cedric Poyet, Benjamin Pradère, Michael Rink, Florian Roghmann, Paul Sargos, Roland Seiler, Francesco Soria, Malte W. Vetterlein, Evanguelos Xylinas

**Affiliations:** a Urology Department, Bichat-Claude Bernard Hospital, Assistance-Publique Hôpitaux de Paris, Paris University, Paris, France; b Urology Unit, Department of Neurosciences, Reproductive Sciences and Odontostomatology, Federico II University of Naples, Naples, Italy; c Urology Department, Helios University Hospital Wuppertal, University of Witten/Herdecke, Germany; d Urology Department, Erasme Hospital, Université Libre de Bruxelles, Brussels, Belgium; e Urology Department, München Klinik Bogenhausen, Munich, Germany; f Urology Department, European Institute of Oncology, Milan, Italy; g Urology Department, Netherlands Cancer Institute-Antoni van Leeuwenhoek Hospital, Amsterdam, The Netherlands; h Department of Urology and Oncological Urology, Wrocław Medical University, Wrocław, Poland; i Urology Department, Careggi Hospital, University of Florence, Florence, Italy; j Urology Department, Luzerner Kantonsspital, Lucerne, Switzerland; k Department of Medical Oncology, Fondazione IRCCS Istituto Nazionale dei Tumori, Milan, Italy; l Urology Department, Sheffield Teaching Hospitals NHS Trust, Sheffield, UK; m Urology Department, University Hospital Zürich, University of Zürich, Zürich, Switzerland; n Urology Department, University Hospital of Tours, Tours, France; o Urology Department, University Medical Center Hamburg-Eppendorf, Hamburg, Germany; p Urology Department, Ruhr-University Bochum, Marien Hospital, Henre, Germany; q Division of Radiation Oncology, Department of Oncology, McGill University, Montreal, QC, Canada; r Department of Urology, University Hospital Bern, Bern, Switzerland; s Urology Division, Department of Surgical Sciences, University of Studies of Torino, Turin, Italy

**Keywords:** Upper tract urothelial carcinoma, Single intravesical postoperative instillation, Chemotherapy, Intravesical recurrence, Radical nephroureterectomy

## Abstract

**Background:**

Level 1 evidence supports the administration of single postoperative intravesical chemotherapy (pIVC) following radical nephroureterectomy (RNU) for upper tract urothelial carcinoma (UTUC), in order to decrease intravesical recurrence risk.

**Objective:**

The Young Academic Urologist Urothelial Cancer Group aimed to investigate the use of pIVC in daily practice among European colleagues.

**Design, setting, and participants:**

An online survey was shared with European Association of Urology Section of Oncological Urology (ESOU) 2017 participants via e-mail. Submissions were accepted from April to June 2017. The topics for 15 questions of this survey included the habit of delivering pIVC, the choice of drug, its dosage, related doubts or concerns, reasons not to perform pIVC, knowledge of the evidence, and surgical preferences for RNU.

**Outcome measurements and statistical analysis:**

Survey software was used for analyses. Logistic regression analyses were used to investigate the association between surgeons’ experience and caseloads with pIVC utilization.

**Results and limitations:**

Overall, 127 responses were collected (11.6%). About half of the participants (47%) regularly administered pIVC following RNU. The drug most commonly utilized was mitomycin (85%); 82% adhered to the standard dosage of 40 mg. Different administration protocols were adopted: ≤48 h (39%), 7–10 postoperative days (35%), >10 d (11%), and intraoperatively (10%). The evidence was supported by prospective randomized clinical trials for only 65% of responders. Among interviewees who did not deliver pIVC, the most commonly reported reasons were lack of supporting data (55%), fear of potential side effects (18%), and organizational hurdles (15%).

**Conclusions:**

Our research highlights the limited use of pIVC following RNU for UTUC, raising the question of how the compliance with level 1 evidence in the urological community may be promoted.

**Patient summary:**

Level 1 evidence supports the administration of single postoperative intravesical chemotherapy (pIVC) following radical nephroureterectomy (RNU) for upper tract urothelial carcinoma (UTUC), in order to decrease intravesical recurrence risk. The Young Academic Urologist Urothelial Cancer Group aimed to investigate the use of pIVC in daily practice among European colleagues. Our research highlights the limited use of pIVC (47%) following RNU for UTUC, raising the question of how the compliance with level 1 evidence in the urological community may be promoted.

## Take Home Message

Our research highlights the limited use of postoperative intravesical chemotherapy following radical nephroureterectomy for upper tract urothelial carcinoma, raising the question of how the compliance with level 1 evidence in the urological community may be promoted.

## 1 Introduction

Bladder cancer detection after radical nephroureterectomy (RNU) for primary upper tract urothelial carcinoma (UTUC) is an event as frequent as it is underevaluated. Intravesical recurrence (IVR) is experienced by 22–47% of patients, most of them within the 1st year of surgery [1], [2]. Although it does not appear to impact survival outcomes significantly, there are several implications that deserve careful evaluation [3], [4]. Increased costs, additional surgeries with related risks, progression of relapses to muscle-invasive bladder cancer, and impairment of contralateral renal function (in patients with already a solitary kidney), but above all waste of time and further suffering for patients, are just some theoretical consequences.

The origin of IVR is still debated—whether monoclonal or polyclonal (expression of descendant seeding or panurothelial defect, respectively) [5]. However, patient-, tumor-, and treatment-specific risk predictors have been identified [5].

Standard UTUC patient management is RNU with bladder cuff excision (BCE), regardless of tumor localization (whether pelvic or ureteral) [1], [6]. Additionally, single postoperative intravesical chemotherapy (pIVC) is delivered to lower the bladder cancer recurrence rate. Two prospective randomized clinical trials and a meta-analysis provide level 1 evidence for safety and efficacy of pIVC [3], [7], [8]. Accordingly, European guidelines strongly recommend delivery of pIVC following RNU [9]. Nonetheless, there are huge discrepancies in the behavior of healthcare professionals, due to more or less well-founded doubts and concerns, or in some cases, poor knowledge of the evidence [10].

The aim of our research was to investigate the use of pIVC in daily practice among the European Association of Urology (EAU) Section of Oncological Urology (ESOU) meeting participants.

## 2 Materials and methods

The Young Academic Urologist (YAU) Urothelial Cancer Group designed the research and developed a questionnaire, promoting it through an online survey. The widely used investigation tool SurveyMonkey.com was used. Fifteen questions were shared with all the ESOU 2017 participants (*n* = 1053; Table 1).

**Table 1:** Survey questionnaire

**Q1**	How do you typically perform the renal portion of RNU?
**Q2**	How do you manage the distal ureter and bladder cuff?
**Q3**	Do you perform a single pIVC after RNU?
**Q4**	If you do not perform single pIVC, why?
**Q5**	Does the prior history of bladder instillations for UBC influence your practice regarding pIVC after RNU?
**Q6**	Does the administration of pIVC after RNU affect the timing of follow-up intervals in your practice?
**Q7**	What is the highest level of evidence for pIVC after RNU?
**Q8**	What kind of agent do you use for chemotherapy?
**Q9**	What dosage do you use in your practice?
**Q10**	Do you perform a cystogram/cystography to ensure closure after BCE?
**Q11**	When do you perform pIVC during the postoperative period?
**Q12**	About what side effects or complications do you counsel patients?
**Q13**	Who do you primarily consider a candidate for pIVC?
**Q14**	Does the possible administration of adjuvant systemic therapy for HR disease after RNU affect the administration of pIVC in your practice?
**Q15**	Does previous administration of any neoadjuvant systemic therapy affect the administration of pIVC in your practice?

BCE = bladder cuff excision; HR = high risk; pIVC = postoperative intravesical chemotherapy; RNU = radical nephroureterectomy; UBC = urothelial bladder cancer.

The survey was available from April to June 2017. The field of investigation ranged from specific surgical preferences to the habit of delivering pIVC. On the latter aspect, participants were asked to answer questions relating to drugs, dosage, doubts and concerns, attitudes, and knowledge of evidence. Survey participation was voluntary and anonymous, with no incentives given. The system assigned a unique random identification number to each respondent, so as to guarantee only one contribution per participant. Only those who responded to all required questions were included in the study (excluded *n* = 189). The analyses that followed were conducted with the survey software. Univariate logistic regression analyses were used to investigate the association between surgeons’ experience and caseloads with pIVC utilization.

## 3 Results

### 3.1 Survey demographics

The survey was distributed to the ESOU participants (*n* = 1053). Among them, an overall complete response rate of 11.6% was observed (*n* = 127). All participants performed the RNU procedure for UTUC management. Overall, 40% were in practice for <10 yr and 88% were performing <10 procedures per year.

### 3.2 Surgical management approaches

**Fig. 1:**
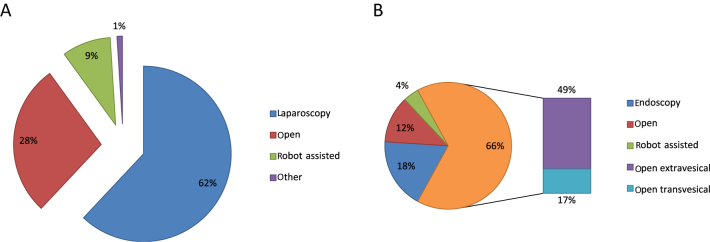
How respondents manage the (A) renal portion and (B) bladder cuff excision of radical nephroureterectomy.

For the renal portion of RNU, most responders (62%) routinely use a conventional laparoscopic approach, including 55% performing it transperitoneally and 7% retroperitoneally. Of the participants, 28% prefer an open approach and 9% robot-assisted laparoscopy (Fig. 1A).

BCE is handled openly by most of participants interviewed (66%, with 49% extravesically and 17% transvesically). Of the responders, 18% choose the endoscopic technique (transurethral resection of the ureteral ostium), 12% laparoscopy, and 4% the laparoscopic robot-assisted approach (Fig. 1B).

### 3.3 Utilization patterns of pIVC

Of the respondents, 47% regularly administered single pIVC following RNU. Moreover, 60% of participants believe all UTUC patients undergoing RNU to be potential candidates for single pIVC; conversely, the decision parameters to instill pIVC were high-grade carcinoma (36%), organ-confined disease (20%), histologically negative lymph nodes (13%), and low-grade carcinoma (7%). Of the participants, 43% did not administer pIVC in case of a previous history of urothelial bladder cancer treated with intravesical therapies.

Neither the number of years in practice (cut-off of <10 or >10 yr) nor the RNU case volume (cut-off of fewer or more than five procedures per year) was significantly associated with pIVC utilization (*p* = 0.42 and *p* = 0.42, respectively).

The habit of administering pIVC was influenced neither by the use of a neoadjuvant systemic therapy for 66% of the responders, nor by the administration of an adjuvant planned systemic chemotherapy for 62% of the responders.

### 3.4 Knowledge patterns of pIVC

**Fig. 2:**
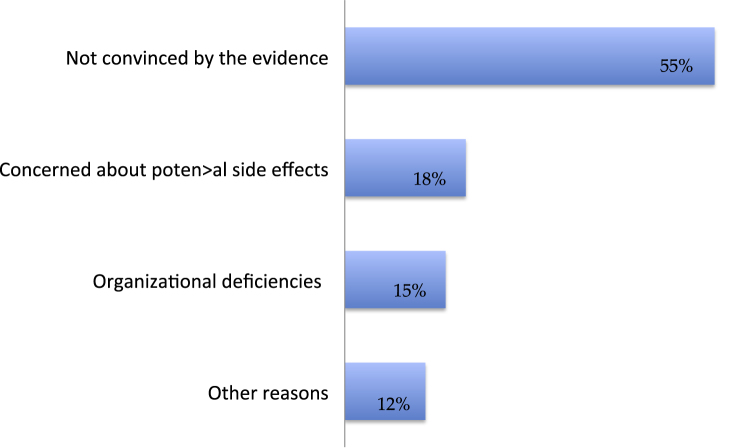
Reasons why respondents declare not to administer pIVC. pIVC = postoperative intravesical chemotherapy.

Up to 68% of the responders were not fully aware of the evidence supporting pIVC administration. More than a third of the responders thought that guideline recommendation was based on pooled retrospective analyses (20%) or experts’ opinion (15%). Moreover, a lack of supporting evidence was the number one reason for not administrating pIVC (55%); fear of the potential side effects (18%) and organizational deficiencies (15%) were the other expressed concerns (Fig. 2).

### 3.5 Instillation practices for pIVC

With regard to the choice of intravesical chemotherapy, 85% used mitomycin-C as the drug of choice (82% at the standard dose of 40 mg) and 10% doxorubicin (50 mg).

**Fig. 3:**
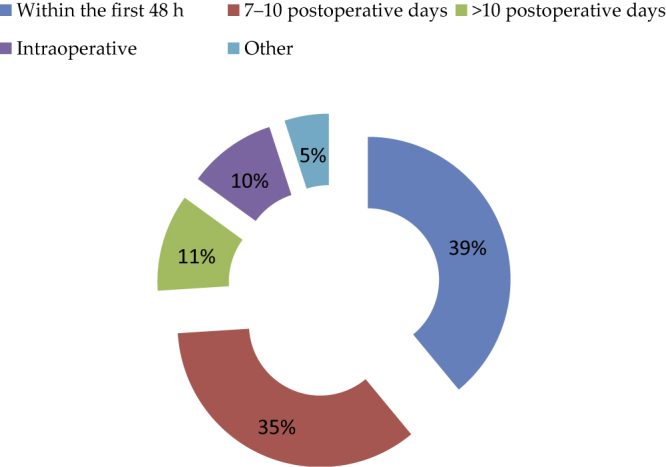
Administration protocols routinely adopted by the survey participants.

Different administration protocols were adopted: first 48 h (39%), 7–10 postoperative days (35%), >10 d (11%), and intraoperatively (10%; Fig. 3). Of the participants, 33% routinely performed a cystogram prior to pIVC administration.

Regarding specific counseling for patients undergoing pIVC, 85% of the responders counsel for local irritative symptoms, 38% for the risk of peritonitis in case of chemotherapy extravasation, and 18% for bladder spasms.

## 4 Discussion

Medical guidelines should form the clinical practice pivot for healthcare professionals. Expression of modern evidence-based medicine and guidelines aim to standardize the healthcare on high-quality standards, while reducing the potential risks for patients, care providers, medical insurers, and health planners. Only by starting with adequate scientific knowledge, it is possible to narrow down to a single patient’s management, considering individual features. Nowadays, prompt application of guideline recommendations cannot rely only on professional updating, but should inevitably involve the entire system of dissemination and advertising currently available. Social media seem to respond incredibly well to this need [11], [12]. To analyze behavioral and probing discrepancies between recommended and actually adopted practices, surveys are useful, quick, and direct means. Our research confirms the topicality of the point addressed.

In a scenario where the optimal management of UTUC patients is still lacking, there are large differences in clinical practice among European oncological urologists. Level 1 evidence for safety and efficacy supports the use of pIVC following RNU for UTUC [3], [7], [8]. However, only 65% of the survey participants demonstrated adequate knowledge of the topic and even less than half (47%) regularly delivered pIVC. Confusion also appears to be increased by the lack of a validated administration protocol (regimen, timing, etc.). Our study showed how this gap generated further daily practice discrepancies. If similar percentages of respondents choose an administration within 48 h or 7–10 d (39% and 35%, respectively), there are also those who prefer to perform it intraoperatively (10%) or even >10 d after surgery (11%). The broad following of monoclonal hypothesis, supported by molecular studies, as well as the clinical detection that after RNU if, on the one hand, 22–47% of patients experience IVR, on the other hand, only 2–6% will harbor contralateral UTUC, highlights how the correct delivery time is not irrelevant [1], [13]. In fact, after 7–10 d, the descended neoplastic cells would have had time to implant in the bladder. The two trials cited from the European guidelines also adopt different approaches. O’Brien et al [3] performed pIVC at catheter removal in the European ODMIT-C trial (about 7 d after surgery); on the contrary, Ito et al [7] preferred to act earlier, within 48 h, in a Japanese trial. The former group motivated the choice by postulating that although not preventing a possible neoplastic implant, it could still have avoided progression to significant new tumors [3]. Furthermore, with regard to the drug administered, the ODMIT-C trial chose mitomycin (40 mg in 40 ml saline), whereas the THP trial chose pirarubicin (30 mg in 30 ml saline) instead [3], [7]. In addition to the lack of knowledge (or lack of belief) of the available evidence and open questions on practical delivery, another important concern among urologists are the potential side effects associated with pIVC administration.

Among the reasons reported to influence the decision for pIVC, 18% of the responders reported being concerned about potential side effects. Certainly, this aspect is well understandable. In fact, if drug extravasation can provoke a bother irritative symptomatology when affecting the retroperitoneal space, the consequences can be dramatic, potentially lethal, if there is an intraperitoneal involvement [3]. However, although cystography can help evaluate impaired bladder closure, our data showed that only 33% of participants use it routinely. In addition, 15% of participants affirm to be limited by organizational shortcomings. This topic should deserve wider discussion. As early as 2014, an American national survey involving the members of the Society of Urologic Oncology highlighted this issue [10]. Despite the ODMIT-C trial recently being published back then, authors were dissatisfied with the circulation and acquisition of the emerging evidence, finding that only about half of the respondents (51%) routinely performed pIVC following RNU [3]. Even at that time, the main barrier was the lack of knowledge of the data supporting its use. After some time and with subsequent publication of the THP trial, our results confirm that the attitude of the European urological community has not changed significantly [7]. Certainly, these two studies have not evaluated the same population, but nevertheless offer a realistic scenario. These considerations must lead us to reflect on the importance of scientific diffusion, which now, more than ever, has the means and possibilities to reach anyone.

While being aware of the theoretical and practical validity of our observations, we are equally aware of the research limits. The response rate was low (11.6%). The adoption of a guideline recommendation may need time; our research represents a snapshot analysis 4 yr after the first appearance of pIVC recommendation in the EAU guidelines. It could be interpreted as a measure of the guideline adoption curve. The number of questions was deliberately limited as much as possible, to increase the compliance of respondents. Consequently, the 15 items selected, even though they made the questionnaire quick and easy, inevitably limited the field of investigation. Participants’ demographics and professional data were not collected and may have impacted the results of our survey. Finally, the questionnaire was designed and implemented entirely by the YAU Urothelial Cancer Group, without any external validation.

## 5 Conclusions

Despite level 1 evidence supporting pIVC administration after RNU for UTUC, large differences in clinical practice among the European oncological urologists were recorded. Less than half (47%) delivered pIVC regularly, and one-third (35%) ignored the evidence. The lack of a validated administration protocol promoted the adoption of heterogeneous approaches in terms of pIVC administration and regimen. A common effort in order to standardize the pIVC management, and even more generally for enhancing the dissemination of evidence, is strongly needed.

  ***Author contributions:*** Evanguelos Xylinas had full access to all the data in the study and takes responsibility for the integrity of the data and the accuracy of the data analysis.

  *Study concept and design:* Xylinas, von Rundstedt.

*Acquisition of data:* Xylinas, von Rundstedt.

*Analysis and interpretation of data:* Xylinas.

*Drafting of the manuscript:* Dobé, Califano, von Rundstedt, Xylinas.

*Critical revision of the manuscript for important intellectual content:* Albisinni, Aziz, Di Trapani, Hendricksen, Krajewski, Mari, Moschini, Necchi, Noon, Poyet, Pradère, Rink, Roghmann, Sargos, Seiler, Soria, Vetterlein.

*Statistical analysis:* Xylinas.

*Obtaining funding:* None.

*Administrative, technical, or material support:* None.

*Supervision:* Xylinas.

*Other:* None.

  ***Financial disclosures:*** Evanguelos Xylinas certifies that all conflicts of interest, including specific financial interests and relationships and affiliations relevant to the subject matter or materials discussed in the manuscript (eg, employment/affiliation, grants or funding, consultancies, honoraria, stock ownership or options, expert testimony, royalties, or patents filed, received, or pending), are the following: None.

  ***Funding/Support and role of the sponsor*:** None.

## CRediT authorship contribution statement

**Tom-Régis Dobé:** Conceptualization, Writing - original draft. **Gianluigi Califano:** Conceptualization, Writing - original draft. **Friedrich-Carl von Rundstedt:** Conceptualization, Methodology, Data curation, Writing - original draft, Supervision. **Idir Ouzaid:** Conceptualization, Writing - review & editing. **Simone Albisinni:** Conceptualization, Writing - review & editing. **Atiqullah Aziz:** Conceptualization, Writing - review & editing. **Ettore Di Trapani:** Conceptualization, Writing - review & editing. **Kees Hendricksen:** Conceptualization, Writing - review & editing. **Wojciech Krajewski:** Conceptualization, Writing - review & editing. **Andrea Mari:** Conceptualization, Writing - review & editing. **Marco Moschini:** Conceptualization, Writing - review & editing. **Andrea Necchi:** Conceptualization, Writing - review & editing. **Aidan P. Noon:** Conceptualization, Writing - review & editing. **Cedric Poyet:** Conceptualization, Writing - review & editing. **Benjamin Pradère:** Conceptualization, Writing - review & editing. **Michael Rink:** Conceptualization, Writing - review & editing. **Florian Roghmann:** Conceptualization, Writing - review & editing. **Paul Sargos:** Conceptualization, Writing - review & editing. **Roland Seiler:** Conceptualization, Writing - review & editing. **Francesco Soria:** Conceptualization, Writing - review & editing. **Malte W. Vetterlein:** Conceptualization, Writing - review & editing. **Evanguelos Xylinas:** Conceptualization, Methodology, Data curation, Supervision.
